# The value of ascitic neutrophil gelatinase‐associated lipocalin in decompensated liver cirrhosis with spontaneous bacterial peritonitis

**DOI:** 10.1002/jcla.23247

**Published:** 2020-02-26

**Authors:** Hua Liu, Ping Zhu, Caiyun Nie, Qing Ye, Yanying Gao, Huaiping Liu, Guoju Pang, Tao Han

**Affiliations:** ^1^ Department of Hepatology The Third Central Clinical College of Tianjin Medical University Tianjin China; ^2^ Department of Hepatology The Third Central Hospital of Tianjin Tianjin China; ^3^ Tianjin Key Laboratory of Extracorporeal Life Support for Critical Diseases Tianjin China; ^4^ Tianjin Institute of Hepatobiliary Disease Tianjin China; ^5^ Artificial Cell Engineering Technology Research Center Tianjin China; ^6^ Department of Oncology The Affiliated Cancer Hospital of Zhengzhou University Zhengzhou China; ^7^ Department of Oncology Henan Cancer Hospital Zhengzhou China; ^8^ Department of Clinical Laboratory The Third Central Hospital of Tianjin Tianjin China

**Keywords:** ascites, biomarker, liver cirrhosis, neutrophil gelatinase‐associated lipocalin, spontaneous bacterial peritonitis

## Abstract

**Background:**

Spontaneous bacterial peritonitis (SBP) is one of the most critical complications of decompensated liver cirrhosis. This study aimed to assess whether ascitic neutrophil gelatinase‐associated lipocalin (NGAL), a reliable inflammation biomarker, can be used to detect SBP in decompensated cirrhosis patients and to predict mortality from decompensated cirrhosis‐related SBP.

**Methods:**

This study included 204 hospitalized patients with ascites of decompensated liver cirrhosis and follow‐up of 28 days. We measured ascitic NGAL levels by the latex‐enhanced immunoturbidimetric method. Simultaneously, we observed the patterns of ascitic NGAL levels in the SBP group after 7 days of anti‐infection treatment with third‐generation cephalosporins.

**Results:**

The ascitic NGAL levels significantly increased in the SBP group compared with that in the non‐SBP group, 111(83.9, 178) ng/mL vs 48(35.4, 63) ng/mL*, P* < .001. Likewise, the ascitic NGAL levels of SBP were higher than non‐SBP with or without renal dysfunction. There was a positive relationship between ascitic NGAL and ascitic polymorphonuclear (PMN) leukocyte and a negative relationship between ascitic NGAL and ascitic albumin in the SBP group. An ascitic NGAL cutoff of 108.95 ng/mL was used for predicting a poor prognosis for SBP patients. Ascitic NGAL and the model for end‐stage liver disease score were independent risk factors in decompensated liver cirrhosis patients with SBP through multivariate Cox regression. A dynamic trend of ascitic NGAL in SBP patients was consistent with the clinical prognosis.

**Conclusion:**

Ascitic NGAL may not only be a biomarker for monitoring SBP but also a predictor for more severe outcomes in decompensated cirrhosis‐related SBP.

## 1 INTRODUCTION

Spontaneous bacterial peritonitis (SBP) often occurs with decompensated liver cirrhosis, and it is characterized by abdominal infection with a high recurrence rate. Once SBP occurs, inflammation may quickly promote the progress of liver and renal dysfunction, even leading to death.^1^ Unfortunately, there is a lack of unique clinical manifestations for SBP. Only one‐third of patients have typical abdominal symptoms and peritoneal irritation, and some patients show intractable ascites and hepatic encephalopathy as the first stage.^2^ Therefore, the best time for antibiotic therapy is missed in the early stages of SBP for some patients, affecting the prognosis. At present, clinical diagnosis of SBP is still established on the presence of polymorphonuclear leukocyte (PMN) ≥ 250 cells/mm^3^ in ascites. Neutrophil gelatinase‐associated lipocalin (NGAL), a member of the lipocalin family, is a low molecular weight secretion protein originally found in activated neutrophils.^3^ There is growing evidence that urinary NGAL can mirror the kidney function damage of cirrhotic patients with acute kidney injury (AKI).^4^, ^5^ Intriguingly, Huelin et al^6^ discovered that urinary NGAL could differentiate the classification of acute tubular necrosis; continuous elevated NGAL is associated with the progression of AKI, which was a predictive factor of 28‐day mortality in the latest research. Although many studies have focused on the relationship between NGAL and AKI, there are relatively few studies on NGAL, especially ascitic NGAL and SBP, another important complication of cirrhosis. Previous research has shown that ascitic NGAL could be suitable for monitoring bacterial peritonitis in emerging non‐malignant ascites.^7^ Inflammation can affect NGAL levels, which in turn can change the inflammatory response and modulate oxidative stress. Animal experiments confirmed the correlation between NGAL and interleukin‐6 levels during sepsis.^8^

The aim of the present study was to investigate the levels of ascitic NGAL in patients with decompensated liver cirrhosis‐related SBP, and whether values of ascitic NGAL can be used to screen for SBP, for measuring the disease dynamics, and for predicting the prognosis of SBP patients.

## 2 MATERIALS AND METHODS

A total of 204 consecutive patients with decompensated liver cirrhosis were included in this study from February 2012 to June 2017 at the Tianjin Third Central Hospital, and data were collected from all patients at the same time.

The diagnosis of decompensated liver cirrhosis was based on the results of clinical and biological examinations and ultrasound and other imaging techniques. SBP was diagnosed on the basis of PMN ≥ 250 cells/mm^3^ and/or positive ascitic fluid culture.^9^ Every enrolled patient received a diagnostic paracentesis at admission for definite diagnosis of SBP. Suspected SBP patients with PMN < 250 cells/mm^3^ required another diagnostic paracentesis within 48 hours. The patients with pre‐existing renal disease, presence of AKI at the time of hospitalization, renal replacement therapy, secondary peritonitis, and malignant diseases were excluded. Baseline variables of all the patients at admission were observed and record included age, sex, etiology of cirrhosis, serum albumin (Alb), alanine aminotransferase (ALT), total bilirubin (TBIL), white blood cells (WBC), percent of neutrophils (N%), platelets (PLT), international normalized ratio (INR), sodium (Na), creatinine (Cr), and procalcitonin (PCT). Ascites detection included ascitic fluid specific gravity, ascitic albumin quantification, polymorphonuclear (PMN), and ascitic culture. In addition, the model for end‐stage liver disease (MELD), Child‐Turcotte‐Pugh (CTP), and serum ascites albumin gradient (SAAG) scores were calculated.

This study was approved by the ethics committee of the The Third Central Hospital of Tianjin. All the patients or their family members provided informed consent.

### 2.1 Ascitic sampling and analysis

The ascitic specimens were sent for examination after paracentesis. The ascite samples were centrifuged at 4°C and 2000 *g* for 10 minutes, and the supernatant was stored at −80°C. NGAL levels were measured by the latexenhanced immunoturbidimetric method (BSBE), which was NGAL in the sample reacted with the supersensitive anti‐NGAL latex particle reagent to form immune complex, and the change in turbidity was proportional to NGAL concentration in the sample.

### 2.2 Statistical analysis

SPSS version 22.0 was used for statistical analyses. Results were expressed as means ± standard deviation (SD) in the normal distributed data, the median (interquartile range) in the abnormal distributed data. Data were analyzed using Student's *t* test and Mann‐Whitney *U* tests for continuous variables and Pearson chi‐square test or Fisher's exact test for categorical variables. Spearman's correlation was applied for the coefficient of correlation between ascitic NGAL and other ascites detection indicators. The area under the receiver operating characteristic curve (AUROC) was used to assess the accuracy of predicting mortality in decompensated liver cirrhosis with SBP. For SBP patients, univariate and multivariate Cox regression analyses were used to estimate the independent risk factors affecting the prognosis. Differences were considered significant when the *P* value was less than .05.

## 3 RESULTS

### 3.1 Baseline clinical characteristics

A total of 204 decompensated cirrhosis patients (141 men, 63 women) were included in this study. The most common etiology of these participants was hepatitis B virus (HBV)‐related cirrhosis (n = 116). The other etiologies included alcoholic cirrhosis (n = 44), hepatitis C virus (HCV)‐related cirrhosis (n = 20), primary biliary cirrhosis (PBC) (n = 12), and cryptogenic cirrhosis (n = 12). There were 64 (31.4%) patients diagnosed with SBP in this study. Ascitic culture was positive in nine patients, including seven *Escherichia coli and* two *Klebsiella pneumoniae*. The baseline characteristics at inclusion of all decompensated cirrhosis patients are shown in Table 1. Compared with the non‐SBP group, ALT, INR, WBC, N%, Cr, and MELD in SBP group were elevated (*P* < .05).

**Table 1:** Comparison of baseline characteristics between SBP group and non‐SBP group

	non‐SBP	SBP	*P* value
n	140	64	
Age (y)	57.31 ± 12.91	59.64 ± 11.95	.640
Sex (n%)			
Male	92 (65.7)	49 (76.6)	.511
Diabetes mellitus n (%)	33 (23.6)	16 (25)	.863
Hepatic encephalopathy n (%)	22 (15.7)	16 (25)	.197
Variceal bleeding n (%)	20 (14.3)	9 (14.1)	.971
CTP n (%)			
B	38 (27.1)	11 (17.2)	.219
C	102 (72.9)	53 (82.8)	.516
MELD	12.07 ± 5.27	18.51 ± 8.22	.009
Alb (g/L)	29.30 ± 4.89	28.41 ± 5.27	.437
ALT (U/L)	29 (17, 48)	39 (21.8, 77)	<.001
TBIL (μmol/L)	45.95 (23.6, 76.5)	42.2 (21.9, 68.2)	<.001
INR	1.53 (1.31, 1.9)	1.61 (1.3, 1.89)	.001
WBC (×10^9^)	4.2 (3.23, 6.82)	5.67 (3.71, 9.58)	<.001
N (%)	68.00 ± 12.97	82.06 ± 8.87	.046
PLT (×10^9^)	66.5 (49, 97)	69 (46, 105)	<.001
Na (mmol/L)	130.44 ± 3.65	131.70 ± 0.88	.354
Cr (μmol/L)	63 (53, 84)	69 (46, 105)	<.001
PCT (ng/mL) (>0.5, n%)	26 (18.6)	15 (23.4)	.515

Note

The values for ALT, TBIL, INR, WBC, PLT, and Cr are median and IQ ranges. The values of the remaining variables are mean ± SD and percentage.

Abbreviations: Alb, albumin; ALT, alanine aminotransferase; Cr, creatinine; CTP, Child‐Turcotte‐Pugh; INR, international normalized ratio of prothrombin time; MELD, model for end‐stage liver disease; N%, percent of neutrophils; Na, natrium; PCT, procalcitonin; PLT, platelets; SBP, spontaneous bacterial peritonitis; TBIL, total bilirubin; WBC, white blood cell.

### 3.2 Ascitic NGAL discriminating SBP in decompensated liver cirrhosis patients

Sixty‐four patients presented SBP, including 45 patients with immediate onset of SBP at admission and 19 patients diagnosed with SBP less than 48 hours after hospital admission. Table 2 shows the statistical differences in baseline ascites tests between patients of the non‐SBP and SBP groups. Ascitic PNM appeared significantly increased, albumin decreased instead in the SBP group (*P* < .05). As expected, the ascitic NGAL level was significantly higher in the SBP group than in the non‐SBP group, 111 (83.9, 178) ng/mL vs 48 (35.4, 63) ng/mL*, P* < .001.

**Table 2:** Comparison of ascites parameters between SBP group and non‐SBP group

	non‐SBP	SBP	*P* value
n	140	64	
Ascites‐specific gravity	1.006 ± 0.087	1.015 ± 0.005	>.05
albumin (g/L)	10.60 ± 2.58	7.53 ± 4.87	.046
PMN (cells/mm^3^)	62.4 (22, 79)	325 (223, 928)	<.001
SAAG	9 (6, 17)	7 (2, 11)	.007
Ascitic NGAL (ng/mL)	48 (35.4, 63)	111 (83.9, 178)	<.001

Note

The values for PMN, SAAG, and ascitic NGAL are median and IQ ranges. The values of ascites‐specific gravity and albumin are mean ± SD.

Abbreviations: NGAL, neutrophil gelatinase‐associated lipocalin; PMN, polymorphonuclear; SAAG, Serum *Ascites* Albumin Gradient; SBP, spontaneous bacterial peritonitis.

### 3.3 Comparison between the ascitic NGAL levels with and without renal dysfunction in both SBP and non‐SBP groups

**Figure 1:**
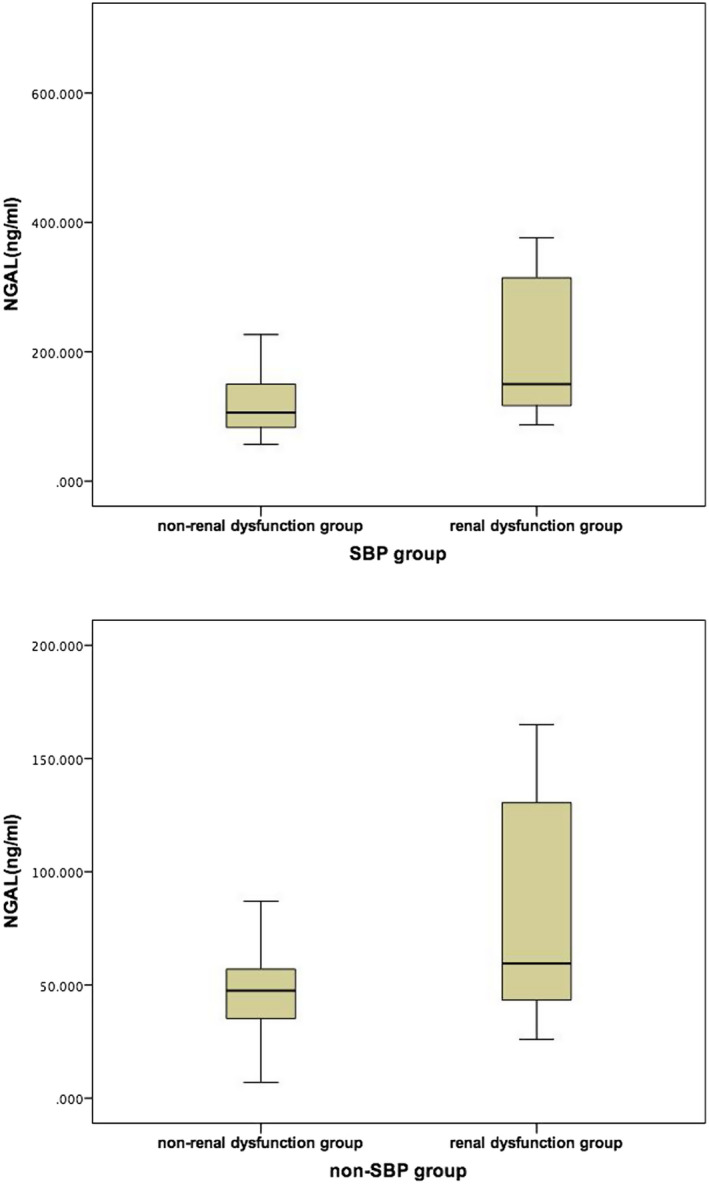
Comparison between the ascitic NGAL levels with and without renal dysfunction in both SBP and non‐SBP groups

In our study, a sub‐analysis of decompensated liver cirrhosis patients showed that 11 patients in the SBP group and 12 patients in the non‐SBP group had renal dysfunction, defined by serum creatinine ≥ 133 μmol/L (1.5 mg/dL). The ascitic NGAL was statistically different in the SBP group with and without renal dysfunction, 150 (99.6, 342) ng/mL vs 106 (83, 150) ng/mL, *P* = .026. Similarly, the non‐SBP group was in line with the SBP group, and there was also a statistical difference between non‐SBP group with and without renal dysfunction, 59.5 (43.35, 130.5) ng/mL vs 47.5 (35.2, 57) ng/mL, *P* = .040, as shown in Figure 1. Even so, the NGAL levels of patients with SBP were still higher than non‐SBP, regardless of renal dysfunction (Table 3).

**Table 3:** Comparison of ascitic NGAL levels with and without renal dysfunction between SBP and non‐SBP groups

	SBP	non‐SBP	*P* value
ascitic NGAL in renal dysfunction group (ng/mL)	150 (99.6, 342)	59.5 (43.35, 130.5)	.000
ascitic NGAL in non‐renal dysfunction group (ng/mL)	106 (83, 150)	47.5 (35.2, 57)	.006

Abbreviations: NGAL, neutrophil gelatinase‐associated lipocalin; SBP, spontaneous bacterial peritonitis.

### 3.4 Relationship between ascitic NGAL and ascitic parameters in SBP patients

In 64 SBP patients who underwent ascitic analysis at admission, we found that baseline ascitic NGAL levels positively correlated with PMN (*r* = .793, *P* < .001) and negatively correlated with ascitic albumin (*r *= −.332, *P* < .001) through the calculation of the relationship between NGAL and other ascitic related parameters (Table 4).

**Table 4:** Correlation coefficients NGAL vs ascitic parameters in decompensated liver cirrhosis patients

Characteristics	*r*‐value	*P* value
Ascites‐specific gravity	.248	>.05
Albumin	−.332	<.001
PMN	.793	<.001
SAAG	.243	>.05

Abbreviations: NGAL, neutrophil gelatinase‐associated lipocalin; PMN, polymorphonuclear; SAAG, Serum *Ascites* Albumin Gradient.

### 3.5 Ascitic NGAL predicts decompensated liver cirrhosis patients with SBP prognosis

**Figure 2:**
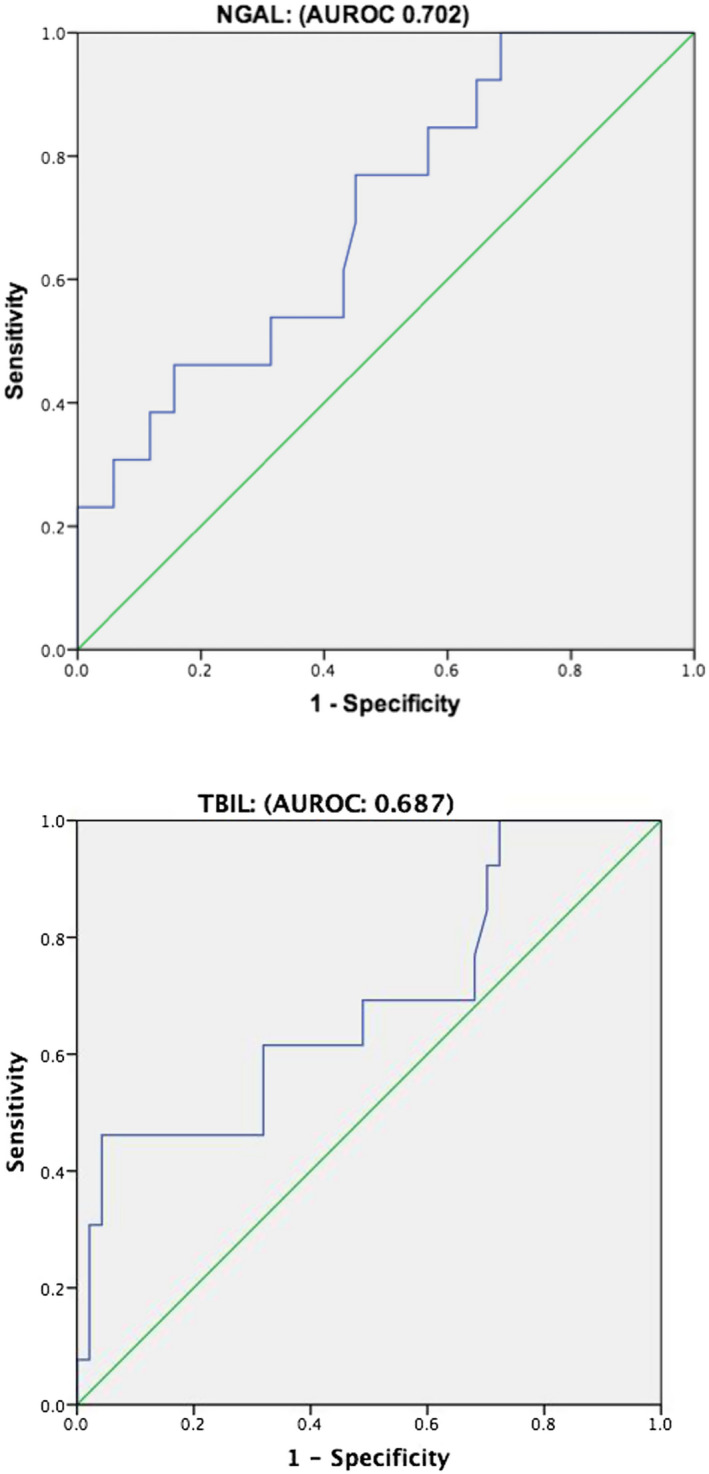
Receiver operator curves calculated for the ascitic NGAL and TBIL in decompensated liver cirrhosis with SBP

In decompensated liver cirrhosis patients of the SBP group, 13 patients died during the 28 days of hospitalization. The ascitic NGAL levels of survivors were significantly lower than that of the non‐survivors, 101 (78.4, 151.5) ng/mL vs 134 (103.05, 446.5) ng/mL, *P* = .025. The ROC curve for NGAL was shown with the AUROC listed. It was obvious that the relevant baseline ascitic NGAL level was the most reliable predictor of mortality in all SBP patients (AUROC 0.702). An ascitic NGAL of 108.95 ng/mL was used as a cutoff value, which with a sensitivity of 76.9% and a specificity of 45.1% (Figure 2), whereas TBIL had a lower AUC value (AUROC 0.687) than NGAL for predicting poor prognosis in SBP patients.

In addition, univariate and multivariate Cox regression analyses were used to further explore the prognostic indicators for the SBP group. In brief, ascitic NGAL and MELD were independent risk factors, which could relevantly predict the prognosis of SBP in decompensated liver cirrhosis patients. The risk ratios (HR) were 1.005 and 1.099, respectively (Table 5).

**Table 5:** Multivariate Cox regression of SBP in decompensated liver cirrhosis patients with SBP

	HR	95% CI	*P*
ascitic NGAL	1.005	1.002‐1.009	.004
MELD	1.099	1.004‐1.204	.042
TBIL	1.001	0.993‐1.009	.751

Abbreviations: CI, confidence interval; HR, risk ratios; MELD, model for end‐stage liver disease; NGAL, neutrophil gelatinase‐associated lipocalin; TBIL, total bilirubin.

### 3.6 Dynamic tendency of ascitic NGAL and prognosis in SBP patients

**Figure 3:**
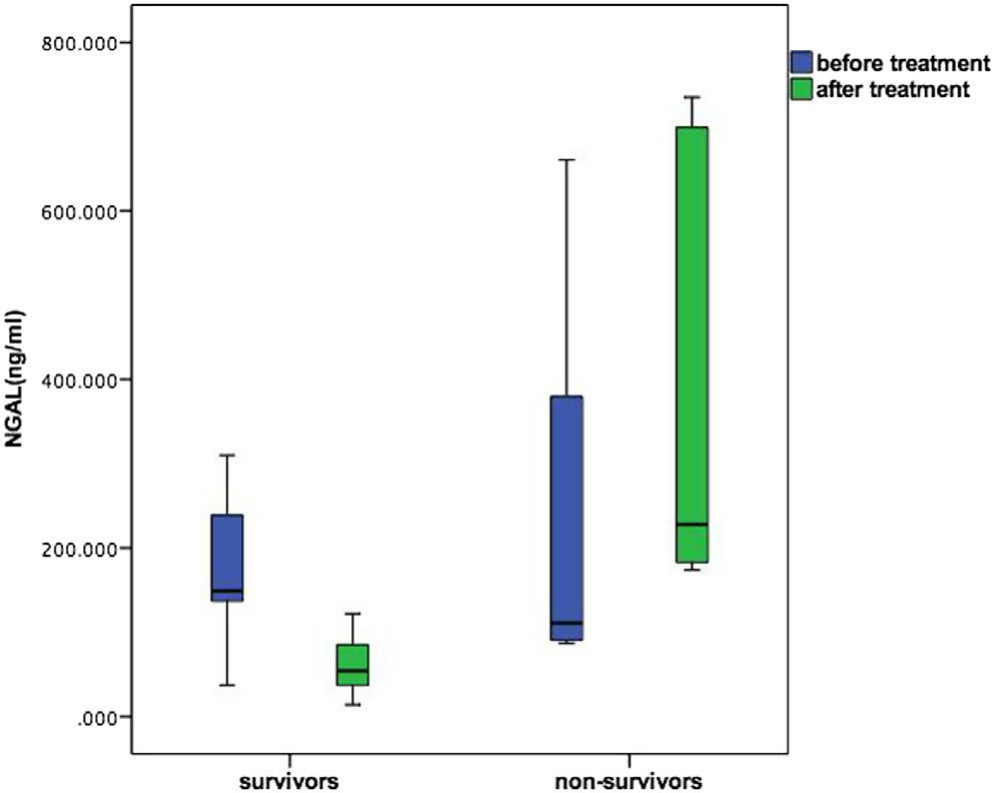
Dynamic changes of ascitic NGAL between survival group and non‐survival group of cirrhosis with SBP after anti‐infection therapy

The changes of ascitic NGAL were dynamically observed in eleven survivors of the SBP group; ascitic NGAL levels declined dramatically after using third‐generation cephalosporins for 7 days, 149 (124, 308.9) ng/mL vs 54.3 (29, 106) ng/mL, *P* = .001. In contrast, even though active antibiotic therapy was administered, the levels of ascitic NGAL were significantly elevated in five non‐survivors of the SBP group, 111 (88.75, 520.1) ng/mL vs 228 (178.5, 717) ng/mL, *P* = .043 (Figure 3).

## 4 DISCUSSION

Nowadays, more and more clinicians pay close attention to co‐infection in decompensated liver cirrhosis because of its prognostic effect; in particular, SBP has attracted much attention because it can cause AKI and increase the mortality rate.^10^, ^11^ Although a PMN count of 250 cells/mm^3^ is widely used as a sensitive diagnostic marker in clinics, there are still some patients with high‐risk factors who cannot be diagnosed according to PMN. Many research groups are attempting to find markers and/or methods for the accurate diagnosis of SBP, including urinary reagent strips^12^, ^13^ and ascitic lactoferrin tests,^14^ but such projects have had diverse outcomes. Therefore, it is critical to improve the survival rate in patients with decompensated liver cirrhosis by early diagnosis, appropriate treatment choice, and close monitoring. Therefore, there is an increasing demand for a marker that can assist in screening for SBP and predict the prognosis of SBP patients.

There had been numerous investigations demonstrated that NGAL, or human lipocalin 2, can play a role in predicting and diagnosing AKI. There is accumulating evidence that NGAL, in combination with other markers, can influence diagnosis of some special renal diseases, such as acute tubular necrosis, diabetic kidney disease, and contrast‐induced nephropathy.^15^, ^16^, ^17^ Considering exocytosis of NGAL from the neutrophilia, rising NGAL levels might not only indicate AKI but also inflammation. In our study, we found that baseline ascitic NGAL levels in the SBP group were significantly more than those in the non‐SBP group in 204 decompensated cirrhosis patients. Moreover, our study demonstrated that ascitic NGAL levels positively correlated with PMN, which is a classic diagnostic marker of SBP. In contrast, the NGAL levels negatively correlated with ascitic protein, so that cirrhotic patients with a ascitic fluid protein below 15 g/L required antibiotics to prevent SBP.^18^ Similarly, in another study using a different detection method, the levels of ascitic NGAL in 29 SBP patients were evidently elevated among the 146 liver cirrhosis patients.^19^

Decompensated liver cirrhosis is characterized by a variety of complications, including various infections. Once inflammation triggers endotoxins, cytokines, oxidative stress, and apoptosis, returning to baseline is much harder.^20^, ^21^, ^22^ Most noteworthy, except for the SBP group, the mortality rate of patients with liver cirrhosis complicated with infection of any type decreased to 31.5%. Therefore, the key to improving the survival of SBP is the early recognition of infection.^23^ In patients whose ascites tests fail to achieve a diagnosis of SBP, the early detection of high‐risk patients with infection still relies on the overall opinion of the evaluating clinician, who needs to use a combination of symptoms, signs, and other laboratory data. NGAL has been proven to be an accurate early diagnostic predictor for AKI.^24^ In addition, in severe acute pancreatitis cases who displayed within 72 hours of symptoms, the levels of urinary and serum NGAL increased significantly from those detected on day 1.^25^ Furthermore, according to a recent report, patients with suspected or confirmed infection in an intensive care unit had higher NGAL levels than non‐infected individuals.^26^ Another major discovery was that if SBP was diagnosed immediately in the hospital or within 48 hours, it was consistent with community acquired SBP, and the baseline ascitic NGAL levels of the SBP patients were increased. A recent publication indicated that baseline urinary NGAL levels can also predict the development of a secondary infection during hospitalization.^27^

As a renal marker, urinary and blood NGAL may be helpful in identifying structural injury and functional damage of the kidney in liver cirrhosis. In the present study, we have shown that, in addition to urine NGAL levels being closely related to AKI, NGAL may be useful to identify the differential types of kidney impairments in cirrhosis.^28^, ^29^ Similarly, an article on the development of acute‐on‐chronic liver failure in patients with liver cirrhosis also discussed baseline serum NGAL as being elevated in renal dysfunction and hepatorenal syndrome in liver cirrhosis.^30^ In our study, increased ascitic NGAL levels were discovered in patients with renal dysfunction in both the SBP and non‐SBP groups. However, despite that, the ascitic NGAL levels of SBP group were still markedly higher than non‐SBP group, with no apparent interference from renal dysfunction.

The presence of portal hypertension and the destruction of intestinal mucosal barriers in patients with cirrhosis, combined with overgrowth of intestinal bacteria, contribute to translocation, which is the main pathogenesis of SBP. It is thought that bacterial DNA could be a promising surrogate marker for bacterial translocation.^20^ As noted, serum NGAL combined with hepcidin is sensitive and specific enough to diagnose bacterial translocation.^31^ This may indicate that NGAL is interrelated with the pathogenesis of SBP. Our study has demonstrated a significant difference between SBP and non‐SBP group with regard to levels of ascitic NGAL. On the other hand, our study also confirmed that trends in the dynamics of NGAL correlate with prognosis in decompensated liver cirrhosis with SBP. It is well known that the liver is a human immune organ, and, as animal experiments proved, hepatocyte‐derived NGAL plays an important role in regulating bacterial infection.^32^

On studying the prognosis of decompensated liver cirrhosis, it was clear that it is affected by many factors. Infection, especially peritonitis, is one of the most important factors in the prognosis of patients with decompensated cirrhosis.^33^ The clinical data of 195 patients showed that Child‐Turcotte‐Pugh classification, ascitic WBC, and ascitic ALB were associated with the occurrence of SBP.^34^ The prognosis depends on the outcome in the battle between host and bacteria. Khan R reported that baseline MELD scores in patients with cirrhosis and ascites may predict SBP recently in the past few days.^35^ In addition, we determined that, compared with TBIL, NGAL may predict mortality with a sensitivity of 76.9% and a specificity of 45.1% in all SBP patients. We also performed Cox regression to prove that ascitic NGAL levels and MELD were independent risk factors in decompensated liver cirrhosis with SBP. It was statistically likely that NGAL was closely related to two mortality‐related complications of cirrhosis, AKI, and SBP.

There are some limitations in our study. On the one hand, the population is relatively small when compared with multicenter clinical trials, and our research can be improved further in the future. On the other, we will conduct a more detailed study of ascitic NGAL in different etiologies of cirrhosis.

To summarize, our study found that, in decompensated liver cirrhosis patients, high levels of ascitic NGAL can play an important role in screening for the occurrence of SBP. In particular, the values of ascitic NGAL can be dynamically measured; different prognoses in SBP patients were associated with the trends in NGAL results. We found an ascitic NGAL of 108.95 ng/mL was the optimum cutoff value for a poor outcome in SBP patients. In addition, ascitic NGAL levels could predict the cirrhosis SBP patients' short‐term prognosis.
